# Second MAFA Variant Causing a Phosphorylation Defect in the Transactivation Domain and Familial Insulinomatosis

**DOI:** 10.3390/cancers14071798

**Published:** 2022-04-01

**Authors:** Christian Fottner, Stefanie Sollfrank, Mursal Ghiasi, Anke Adenaeuer, Thomas Musholt, Arno Schad, Matthias Miederer, Simin Schadmand-Fischer, Matthias M. Weber, Karl J. Lackner, Heidi Rossmann

**Affiliations:** 1 Department of Endocrinology and Metabolism, I Medical Clinic, University Medical Center of the Johannes Gutenberg University Mainz, 55131 Mainz, Germany; christian.fottner@unimedizin-mainz.de (C.F.); matthias.weber@unimedizin-mainz.de (M.M.W.); 2 Institute of Clinical Chemistry and Laboratory Medicine, University Medical Center of the Johannes Gutenberg University Mainz, 55131 Mainz, Germany; stefanie.sollfrank@hotmail.de (S.S.); mursal_ghiasi@hotmail.de (M.G.); anke.adenaeuer@unimedizin-mainz.de (A.A.); karl.lackner@unimedizin-mainz.de (K.J.L.); 3 Clinic of General, Visceral- and Transplantation Surgery, Endocrine Surgery Section, University Medical Center of the Johannes Gutenberg University Mainz, 55131 Mainz, Germany; thomas.musholt@unimedizin-mainz.de; 4 Institute of Pathology, University Medical Center of the Johannes Gutenberg University Mainz, 55131 Mainz, Germany; arno.schad@unimedizin-mainz.de; 5 Department of Nuclear Medicine, University Medical Center of the Johannes Gutenberg University Mainz, 55131 Mainz, Germany; matthias.miederer@unimedizin-mainz.de; 6 Department of Radiology, University Medical Center of the Johannes Gutenberg University Mainz, 55131 Mainz, Germany; simin.schadmand-fischer@unimedizin-mainz.de

**Keywords:** insulinomatosis, insulinoma, hyperinsulinemic hypoglycemia, MAFA, phosphorylation defect

## Abstract

**Simple Summary:**

Adult-onset familial insulinomatosis is a rare disorder with recurrent, severe hypoglycemia caused by multiple insulin-secreting pancreatic tumors. The etiology was unclear until the genetic variant p.Ser64Phe in the MAFA protein, a key coordinator of insulin secretion in pancreatic cells, was defined as the cause in two families. Based on the cases of two sisters with insulinomatosis, we aimed to identify further disease causes. The sequencing of the complete coding regions of the patients’ genomes revealed a second genetic MAFA variant, p.Thr57Arg, as the cause of familial insulinomatosis, linking genetic, clinical, and biochemical analyses from the patients’ family to the pre-described cell culture data. Thus, we confirm a defect in a crucial regulatory region of the MAFA protein as an important cause of a specific hereditary syndrome, which is characterized by insulinomatosis and/or mild hyperglycemia. This study extends the pathophysiological and diagnostic disease concept and verifies the inheritance pattern of familial insulinomatosis.

**Abstract:**

Adult-onset familial insulinomatosis is a rare disorder with recurrent, severe hypoglycemia caused by multiple insulin-secreting pancreatic tumors. The etiology was unclear until the variant p.Ser64Phe in the transcription factor MAFA, a key coordinator of β-cell insulin secretion, was defined as the cause in two families. We here describe detailed genetic, clinical, and family analyses of two sisters with insulinomatosis, aiming to identify further disease causes. Using exome sequencing, we detected a novel, heterozygous missense variant, p.Thr57Arg, in MAFA’s highly conserved transactivation domain. The impact of the affected region is so crucial that in vitro expression studies replacing Thr57 have already been performed, demonstrating a phosphorylation defect with the impairment of transactivation activity and degradation. However, prior to our study, the link to human disease was missing. Furthermore, mild hyperglycemia was observed in six additional, heterozygote family members, indicating that not only insulinomatosis but also MODY-like symptoms co-segregate with p.Thr57Arg. The pre-described MAFA variant, p.Ser64Phe, is located in the same domain, impairs the same phosphorylation cascade, and results in the same symptoms. We confirm MAFA phosphorylation defects are important causes of a characteristic syndrome, thus complementing the pathophysiological and diagnostic disease concept. Additionally, we verify the high penetrance and autosomal dominant inheritance pattern.

## 1. Introduction

Hypoglycemia due to endogenous hyperinsulinism is a rare clinical condition. In newborn and infants, rare monogenetic diseases caused by variants in genes involved in the regulation of insulin secretion (*ABCC8*, *KCNJ11*, *GCK*, *HADHSC*, *INSR*, *GLUD1*, *SLC16A1*, *HNF1A*, *HNF4A*, and *UCP2*) present with severe persistent hypoglycemia [1]. In adults, however, the most common cause of this pathology is either sporadic insulinoma (80%) or insulinoma associated with multiple endocrine neoplasia type I (MEN1) (9–10%) [2,3]. Although insulinomas can be small and sometimes challenging to detect, modern diagnostic and surgical techniques usually allow localization and successful resection today. In the remaining 10% of cases, endogenous pancreatic hyperinsulinism is either due to insulinomatosis or functional β-cell disorders, clinically termed as noninsulinoma pancreatogenous hypoglycemia syndrome (NIPS) and adult nesidioblastosis [4,5]. They are histopathologically characterized by β-cell hypertrophy, islet hyperplasia, and an increase in β-cell mass. Disease pathogenesis is largely unknown and may be different from congenital hyperinsulinemic hypoglycemia, given that so far no distinct genetic causes have been identified in adult patients with NIPS/nesidioblastosis [1,6,7]. However, the observed association with gastric bypass surgery in obese patients suggests that a reactive process possibly unmasks or induces a defect in β-cells, resulting in hyperfunction [8,9].

Insulinomatosis is another rare cause of hyperinsulinemic hypoglycemia in adults and is characterized by the synchronous and metachronous occurrence of small insulinomas, multiple insulinoma precursor lesions, and small proliferative insulin-expressing monohormonal endocrine cell clusters (IMECCs) [10]. Clinically, in parallel to few macrotumors (usually 0.5–1 cm), multiple microtumors are found throughout the entire pancreas, expressing exclusively insulin. Due to the small size and the multicentric occurrence of the tumors, both diagnosis and therapy are clinically challenging. The clinical course of the disease is characterized by early recurrent hypoglycemia requiring repeated surgical intervention. Already in 1977, Tragl et al. were the first to describe a family with multiple adenomas restricted to β-cells and the occurrence of diabetes mellitus in different members of the same family, raising the possibility of a common genetic origin [11]. However, only recently, a disease-causing variant in the V-MAF avian musculoaponeurotic fibrosarcoma oncogene homolog A (MAFA) basic leucine zipper-containing protein has been identified causing insulinomatosis [12]. MAFA belongs to the family of large MAF transcription factors and is expressed in islet β-cells. It is required for postnatal β-cell function and acts as a transactivator of insulin and several genes involved in glucose-stimulated insulin secretion [13,14,15,16,17]. The only clinically relevant MAFA variant known, Ser64Phe, is associated with the autosomal dominant inheritance of either insulinomatosis or diabetes mellitus, documenting the physiological properties of MAFA, both as an oncogene and key islet β-cell transcription factor [12]. In this study, we investigated two sisters with recurrent hyperinsulinemic hypoglycemia for genetic causes. Using whole exome sequencing, bioinformatics, and family analysis, we identified a second, hitherto unreported *MAFA* variant (NM_201589.4: c.170C>G, p.(Thr57Arg)) as a cause of insulinomatosis and mild hyperglycemia in other family members. The exchange of Thr57 results in a phosphorylation defect as it is located in the functionally crucial part of the transactivator domain, and its relevance has already been demonstrated by others.

## 2. Materials and Methods

### 2.1. Patients

**Figure 1:**
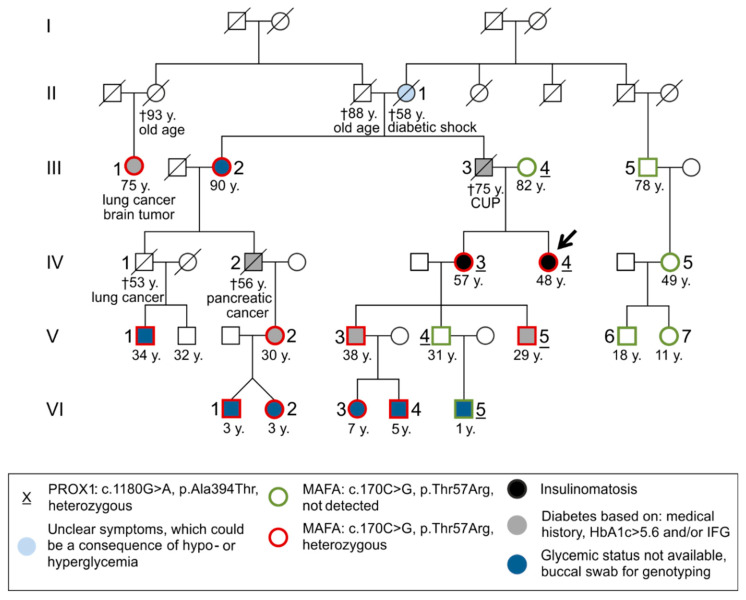
Pedigree, presenting genotype, phenotype and age of the family members. Four generations of the family underwent further molecular genetic (*MAFA* Thr57Arg and *PROX1* Ala394Thr; genotyped individuals marked by the bold border of the symbols) and laboratory testing as well as an evaluation of the medical history. In individuals with the symbol filled in dark blue, only a buccal swab was available for laboratory analysis (III2, V1, and VI1–5), suitable for genotyping but not for metabolic analyses, e.g., the determination of HbA1c. *MAFA* Thr57Arg was detected (symbols with red bold border) in 12 family members (III1–2, IV3–4, V1–3, V5, and VI1–4), 2 with insulinomatosis (IV3–4, arrow points on index case), 4 with an overt diabetes (III1 (based on patient medical history), V2–3 and V5 (HbA1c > 5.6 or impaired fasting glucose (IFG)), and 6 members (4 children below 10 years (VI1–4) and 2 adults (III2 and V1)) whose glycemic status was unclear, because blood samples were not available. Furthermore, diabetes mellitus was known in 2 deceased individuals (III3 and IV2). In 7 family members who did not carry Thr57Arg (symbols with green bold border), neither diabetes nor insulinomatosis has been detected (III4–5, IV5, and V4 + 6–7) or they had an unclear glycemic status (VI5). Subject numbers of the six *PROX1* Ala394Thr carriers are underlined (III4, IV3–4, V4–5, and VI5). The *MAFA* mutation is inherited through the left wing of the pedigree (starting with the left couple in generation I). The *PROX1* variant, on the other hand, was introduced into the pedigree by person III4 and is, thus, inherited only throughout one branch. CUP = cancer of unknown primary; y. = age in years; † = deceased.

Two German sisters suffering from insulinomatosis (detailed case reports for both are provided in the Results section) and their family were evaluated (Figure 1: 17 relatives were genotyped; ten of them also underwent physical examination and blood sampling). Clinical evaluation of the relatives included age, sex, body mass index (BMI), previous illnesses, hospitalizations, allergies, medication, use of nicotine and alcohol, an orienting physical examination and routine clinical chemistry, hematology, coagulation, and endocrine testing (see below). Special attention was paid to the report of symptoms of diabetes mellitus, hypoglycemia, eye diseases (especially hereditary cataract and glaucoma), and malignancy.

Ten samples from German healthy volunteers were analyzed in parallel with the genomic DNA (gDNA) of the two sisters by Next Generation Sequencing to eliminate common local variants and sequencing artifacts.

All laboratory analyses in the patients were performed for diagnostic purposes at the Institute of Clinical Chemistry and Laboratory Medicine and the Institute of Pathology, University Medical Center Mainz. Patients and healthy controls provided explicit consent to the use of their pseudonymized data for research purposes. The studies were designed and executed in accordance with all local legal and regulatory requirements, notably the General Data Protection Regulation (EU 2016/679) and the Declaration of Helsinki, 7th revision.

### 2.2. Routine Laboratory Analyses

Family members were subjected to a set of routine blood analyses: Clinical chemistry analyses (Na^+^, K^+^, Ca^2+^, glucose, creatinine, alanine aminotransferase (ALAT), aspartate aminotransferase (ASAT), alkaline phosphatase, γ-glutamyl transferase, total bilirubin, lipase, α-amylase, C-reactive protein (CRP), albumin, urea-N, phosphate, triglyceride, and total and HDL-cholesterol) were performed on an Architect c8000 system (Abbott, Wiesbaden, Germany); hormone analyses (insulin, pro-insulin, C-peptide, and thyroid-stimulating hormone (TSH)) were performed on an Architect i2000 system (Abbott, Wiesbaden, Germany); whole blood counts were performed on an Advia 2120i Hematology System (Siemens Healthcare GmbH, Erlangen, Germany); coagulation testing (Quick, activated partial thromboplastin time (aPTT), derived Fibrinogen) was performed on an ACL TOP 700 instrument (Instrumentation Laboratory (IL), Munich, Germany); and HbA1c on a Variant II Hemoglobin Testing System (BioRad, Munich, Germany), all using reagents of the respective device manufacturers. The estimated glomerular filtration rate (eGFR) (according to the CKD-EPI formula), LDL-cholesterol (according to Friedewald), and the LDL/HDL ratio were calculated.

### 2.3. Histopathology and Immunohistochemistry

Serial sections from archival pancreatic tissue were subjected to MAFA immunohistochemistry as well as insulin and glucagon staining. The result was evaluated and compared to that of normal human pancreas by an experienced pathologist. To achieve comparability, MAFA immunohistochemistry was performed using the same MAFA antibody (Anti-MafA antibody ab26405, Abcam, Cambridge, UK, RRID: AB_776146; dilution: 1:2000) as Iacovazzo et al. [12] following the manufacturer’s instructions. After deparaffinization, hydration, and heat-induced epitope retrieval, incubation steps were carried out in an Autostainer 480-A (Thermo Fisher Scientific, Dublin, Ireland) using the Dako EnVision™ FLEX HRP/Dab (Agilent, Santa Clara, CA, USA) detection system with signal amplification by EnVision™ FLEX + Rabbit (LINKER) (Agilent, Santa Clara, CA, USA).

Standard staining/immunohistochemisty protocols of the Institute of Pathology (University Medical Center Mainz) were used to detect the neuroendocrine/proliferation markers chromogranin, synaptophysin, insulin, glucagon, and KI-67 in formalin-fixed, paraffin-embedded pancreatic tissue sections from patients IV3 and IV4 following resection.

### 2.4. DNA Extraction and Sequencing

Genomic DNA was isolated from 200 µL whole blood or from buccal swabs OG-675 (Oragene DNA, DNA Genotek, Ottawa, ON, Canada) using the blood/saliva protocol of the QIAamp DNA Mini kit. For primary evaluation of case 1 and 2, genetic variants were detected by whole exome sequencing (WES) as described by Barco et al. [18], except for the hybridization probe set (NimbleGen MedExome, Roche, Pleasanton, CA, USA). Sanger Sequencing (Beckman CEQ8000, Sciex, Darmstadt, Germany; Wenzel et al. [19]) and multiplex ligation-dependent probe amplification (SALSA MLPA Probemix P017, MRC Holland, Amsterdam, The Netherlands) were used for *MEN1* testing prior to WES and to confirm the variants detected by WES. Targeted analysis of family members was performed by Sanger- or pyrosequencing (PyroMark Q96 ID, Qiagen, Hilden, Germany), as described previously [20], with PCR primers (Integrated DNA Technologies, Coralville, Iowa, USA), and the conditions adapted to the current analytics (primers for Sanger sequencing: MAFA_T57R.for ACGACTTCGACCTGATGAAGTTCG, MAFA_T57R.rev CCCCGGCCTGAGACGAGC, PROX1_A394T.for CTGCCATGTCGCAAGTTGTG, PROX1_A394T.rev AACTGGCCATCTGCACATTG; primers for pyrosequencing: MAFA_T57R_PSQ.for 5′ Biotin tagged CGCCAGGCTCGCTGTCCT, MAFA_T57R_PSQ.rev GGCACGGAGGAGCAGGG, MAFA_T57R_PSQ.Rseq ACGGAGGAGCAGGGC, dispensation order TCGTGCTG).

### 2.5. Bioinformatics Analyses

The quality of WES was checked with fastQC (Babraham Bioinformatics) and the sequencing analysis viewer (Illumina, San Diego, CA, USA). The format conversion of the resulting fastq-files, whole-genome alignment (GRCh37/hg19), variant calling, and copy number variation (CNV) detection was performed using NextGENe, version 2.4.1.1 (Softgenetics, State College, PA, USA). Variants were filtered by an in-house Perl-pipeline: All variants present in the ten controls were excluded, as well as variants with a minor allele frequency (MAF) above 0.01 in dbSNP. The remaining variants were reduced by two gene lists: one targeting genes known to cause endocrine tumors (*AIP*, *AP2S1*, *BAP1*, *BRAF*, *CASR*, *CDC73*, *CDKN1A*, *CDKN1B*, *CDKN2B*, *CDKN2C*, *EGLN1*, *EGLN2*, *EPAS1*, *FH*, *GCM2*, *GNA11*, *H3F3A*, *HRAS*, *IDH1*, *KIF1B*, *KRAS*, *MAX*, *MDH2*, *MEN1*, *NF1*, *PTH*, *RET*, *SDHA*, *SDHAF2*, *SDHB*, *SDHC*, *SDHD*, *SLC25A11*, *TMEM127*, *TP53*, *TSC1*, *TSC2*, and *VHL*); and the other targeting genes, which are involved in nutrient sensing, insulin secretion, growth and differentiation of pancreatic β-cells, or known causes for maturity onset diabetes of the young (MODY) as well as syndromic and non-syndromic congenital hyperinsulinism (*ABCC8*, *ADAMTS9*, *ADCY5*, *ADK*, *AKT1*, *AKT2*, *AKT3*, *ALG3*, *ALG6*, *APPL1*, *ARX*, *BCL11A*, *BCL2*, *BLK*, *CACNA1D*, *CAMK1D*, *CDC123*, *CDKAL1*, *CDKN1C*, *CDKN2A*, *CDKN2B*, *CEL*, *CENTD2*, *CREB1*, *DGKB*, *DUSP9*, *ESRRG*, *FOXA1*, *FOXA2*, *FOXO1*, *FTO*, *G6PC2*, *GCG*, *GCK*, *GCKR*, *GIP*, *GIPR*, *GLIS3*, *GLP1R*, *GLUD1*, *GSK3B*, *GSTM1*, *H19*, *HADH*, *HHEX*, *HK1*, *HKDC1*, *HMGA2*, *HNF1A*, *HNF1B*, *HNF4A*, *IGF2*, *IGF2BP2*, *INS*, *INSR*, *IRS1*, *IRS2*, *ISL1*, *ITGB6*, *JAZF1*, *JUN*, *KCNJ11*, *KCNQ1*, *KDM6A*, *KLF11*, *KLF14*, *KMT2D*, *LGR5*, *MAF*, *MAFA*, *MAFB*, *MAPK1*, *MAPK3*, *MAPK8*, *MCAT*, *MEN1*, *MNX1*, *MPI*, *MTNR1B*, *NCOA6*, *NEUROD1*, *NEUROG3*, *NKX2-2*, *NKX6-1*, *NOTCH2*, *OASL*, *ONECUT1*, *P2X7*, *PAX4*, *PAX6*, *PCBD1*, *PCSK1*, *PDLIM5*, *PDX1*, *PGM1*, *PIK3CA*, *PIK3R1*, *PIK3R2*, *PMM2*, *POC1A*, *PPARG*, *PRC1*, *PRKAA2*, *PROX1*, *PTF1A*, *RBMS1*, *SIRT1*, *SLC16A1*, *SLC2A2*, *SLC30A8*, *TCF7L2*, *THADA*, *TLE4*, *TMEM195*, *TP53INP1*, *TSPAN8*, *UCP2*, *VDR*, *WFS1*, and *ZBED3*). Variants detected in both sisters, were considered as potential causes of the disease.

## 3. Results

### 3.1. Case Report

#### 3.1.1. Clinical Case 1; Index Patient (Female, IV4, 48 Years Old at Time of Study)

**Figure 2:**
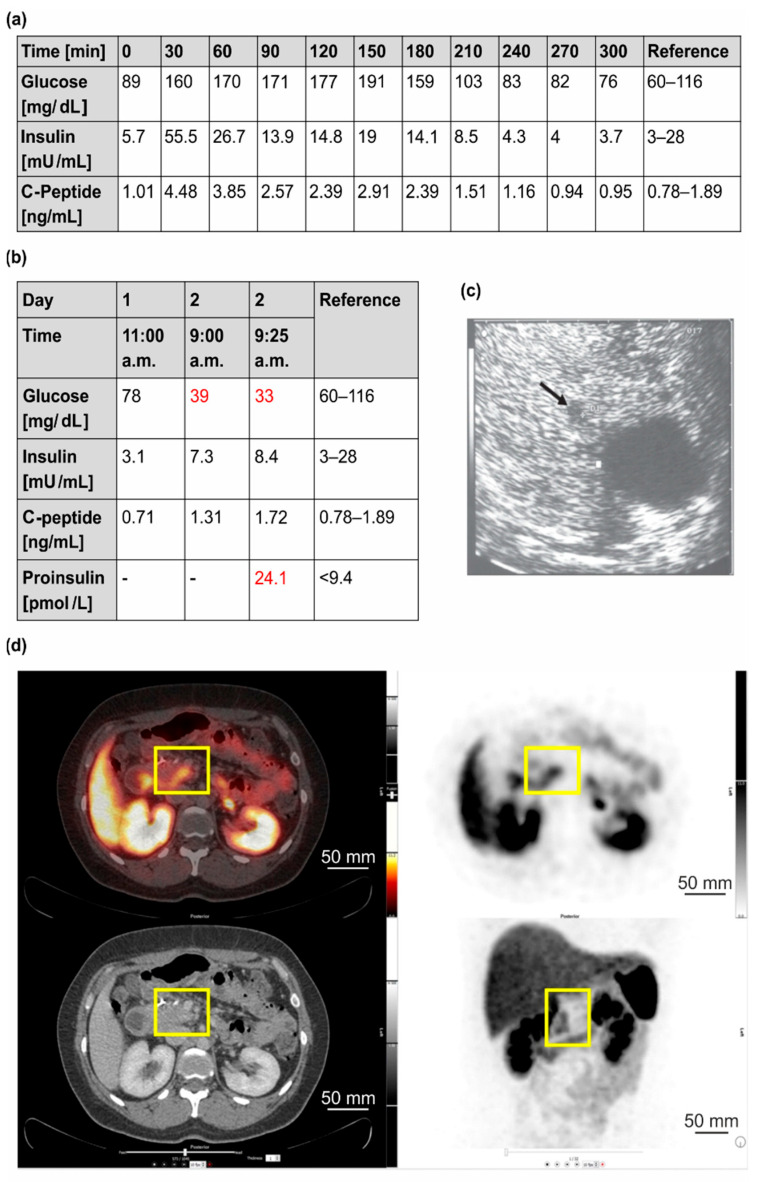
Laboratory tests and diagnostic imaging of case 1 (index patient, IV4). (**a**) Endocrine testing showed unremarkable oral glucose tolerance (prolonged test conditions) without clinical or biochemical signs of hypoglycemia. (**b**) A 72 h fasting test showed marked hypoglycemia after 22 h with a plasma glucose as low as 33 mg/dL and corresponding serum C-peptide, insulin, and proinsulin levels inadequately elevated. (- = not performed). Values outside the reference range are highlighted in red. (**c**) Further imaging with endoscopic ultrasound revealed a single lesion of 4–5 mm. (**d**) Functional imaging with 68Ga-DOTATOC-PET/CT was negative with low and diffuse uptake in the pancreatic head (yellow frames), interpreted as unspecific.

**Figure 3:**
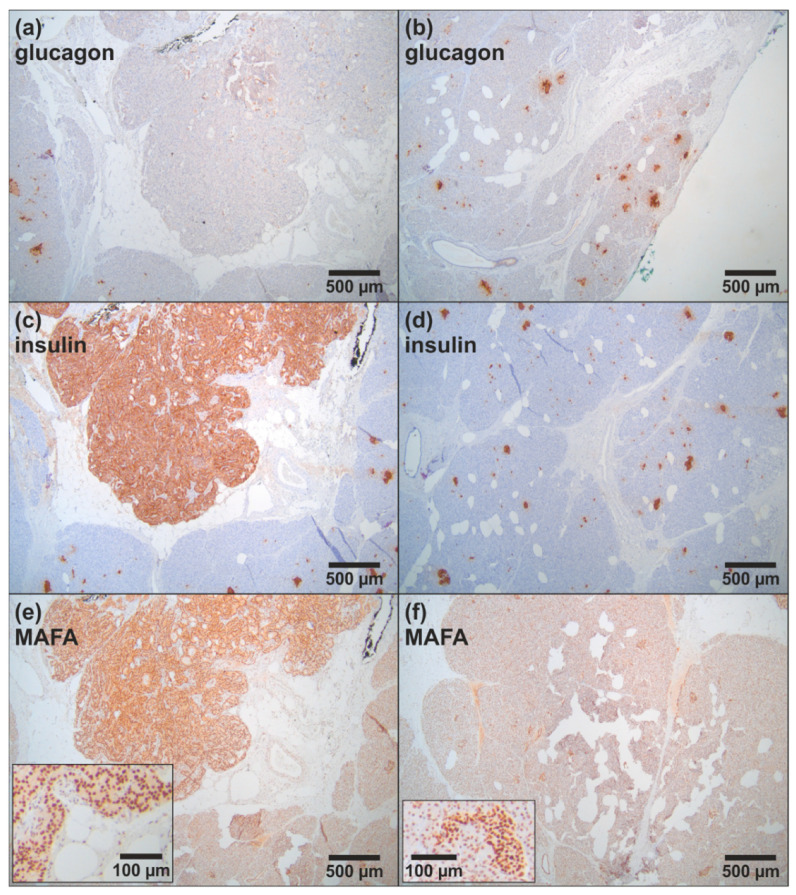
Glucagon, insulin and MAFA immunohistochemistry of case 1 (IV4). Immunohistochemical staining of serial sections of the resected pancreatic specimen of case 1 showing a solitary insulinoma (left panels (**a**,**c**,**e**)) and normal pancreatic tissue with multiple pancreatic islets (right panels (**b**,**d**,**f**)).

Starting at the age of 34, the patient experienced continuous weight gain (>20 kg) and recurrent episodes of severe hunger. An initial medical and psychiatric examination was unremarkable. It has been performed due to a three-hour incident in which the patient had damaged other vehicles with her car but could not remember it afterwards. At the age of 38, a “low blood glucose level” was incidentally detected. Consequently, a 72-h fasting test was initiated and had to be stopped after 4 h due to hypoglycemia (16 mg/dL) with increased insulin and C-peptide levels (data not shown). An endoscopic ultrasound (EUS) of the pancreas showed a 7 mm lesion in the tail of the pancreas (data not shown). The patient received left-sided pancreatic resection with the histological diagnosis of a well-differentiated neuroendocrine tumor (NET) of 0.6 cm that was immunohistochemically positive for chromogranin, synaptophysin, and insulin (NET G1, Ki-67 index 1%, pT1, pN0, L0, V0, Pn0, R0, G1). After surgery, she still complained about hypoglycemia symptoms. Diagnostic work-up revealed persistent hyperinsulinemic hypoglycemia with another solitary lesion of about 5 mm in the pancreatic body (in EUS) (Figure 2a–c). Functional imaging with 68Ga-DOTATOC-PET/CT was negative (Figure 2d). Biochemical and genetic analyses (Sanger sequencing and MLPA of the *MEN1* gene) provided no evidence for *MEN1* variants. Diagnostic laparotomy with intraoperative ultrasound identified two focal lesions that were approximately 5 mm in diameter. Histological examination after partial resection of the pancreatic head confirmed the diagnosis of insulinomatosis with multifocal microadenomas that were monohormonally positive for insulin and mostly <5 mm in size, except for two larger adenomas (NET G1) of 6 and 7 mm in diameter. Since the second surgical intervention, the patient was asymptomatic. After the detection of the germline *MAFA* variant Thr57Arg, MAFA immunohistochemistry was performed on residual archival tissue of the resected pancreatic specimen. MAFA protein expression was found in the nuclei of islet cells as well as in the nuclei of an insulinoma. Although the data should be considered preliminary, at least no obvious difference in *MAFA* expression was found between patient islets and insulinoma and wild-type islets from non-insulinoma patients (Figure 3).

#### 3.1.2. Clinical Case 2; Sister of Clinical Case 1 (IV3, 57 Years Old at Time of Study)

**Figure 4:**
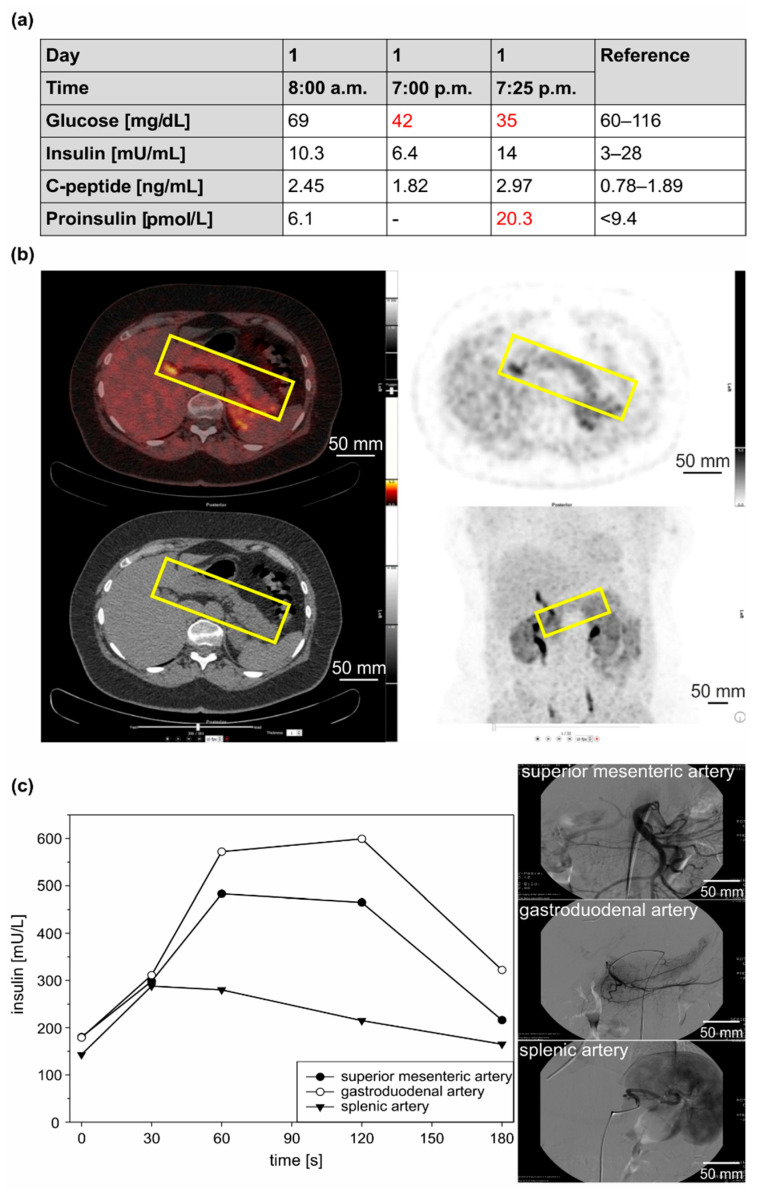
Laboratory tests and diagnostic imaging of case 2 (sister of index patient, IV3). Endocrine testing showed normal prolonged oral glucose tolerance without clinical or biochemical signs of hypoglycemia (data not shown). (**a**) A 72 h fasting test was stopped after 11 h showing inadequately elevated insulin, C-peptide, and proinsulin levels. (- = not performed). Values outside the reference range are highlighted in red. (**b**) Functional imaging with 18F-DOPA-PET/CT showed physiological radionuclide-accumulation in the common bile duct and a diffuse, non-homogeneous uptake in the whole pancreas (yellow frame), judged to be unspecific. (**c**) The selective arterial calcium infusion (SACI)-test showed a 2- to 3-fold increase of insulin secretion after calcium-stimulation of all three arteries (superior mesenteric, gastroduodenal and splenic artery).

Since the age of 45, the patient had suffered from recurrent episodes of fatigue, confusion, blurred vision, and tremors, which improved with food intake. The consequence was continual weight gain (11 kg). At the age of 53, a low blood glucose level (60 mg/dL) was detected when the patient was found to be unconscious after physical stress. Persistent symptoms and the sister’s medical history finally prompted a diagnostic work-up. A prolonged oral glucose tolerance test (oGTT) was unremarkable. A 72 h fasting test was stopped after 11 h with a blood glucose level of 35 mg/dl and clinical signs of hypoglycemia, immediately reversible after intravenous glucose infusion (Figure 4a). The corresponding insulin-, C-peptide- and pro-insulin levels were inappropriately elevated (14 mU/mL, 2.97 ng/mL, and 20.3 pmol/L). Molecular genetic testing for *MEN1* was negative, as were imaging procedures (including magnetic resonance imaging (MRI), EUS, and 68Ga-DOTATOC-PET/CT). Functional imaging with 18F-DOPA-PET/CT (GLP-1-receptor-imaging was not available at that time) showed physiological radionuclide accumulation in the common bile duct and a diffuse, homogeneous uptake throughout the pancreas, although this was judged to be nonspecific (Figure 4b). A 2-fold to 3-fold increase in insulin levels was observed in all three arteries (superior mesenteric, gastroduodenal, and splenic artery) in response to selective arterial calcium infusion (SACI) (Figure 4c). Histological evaluation after a duodenum-preserving resection of the pancreatic head showed multiple small insulin-positive tumorlets with 2–4 mm in diameter, consistent with the diagnosis of insulinomatosis. Since the operation, the patient has been asymptomatic.

### 3.2. Molecular Genetic Testing

#### 3.2.1. Molecular Genetic Testing of the Two Patients and Classification of the Detected Variants According to the ACMG Criteria

**Figure 5:**
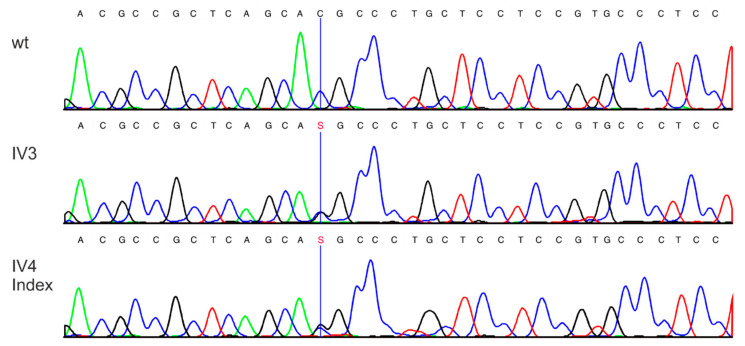
Confirmation of variant NM_201589.3 (*MAFA*): c.170C>G, p.Thr57Arg, in the two affected siblings by Sanger sequencing. wt: healthy control; IV3 corresponds to case 2 (Figure 1); IV4 Index to case 1.

WES in the index patient and her sister and the subsequent selection of the rare variants present in both sisters from the predefined set of 158 genes (Patients and Methods section) revealed two variants, both in heterozygous state: c.1180G>A, p.Ala394Thr in the *PROX1* and c.170C>G, p.Thr57Arg in the *MAFA* gene (Table 1), which were confirmed by Sanger Sequencing (Figure 5). PROX1 and MAFA are both transcription factors involved in β-cell differentiation, although unlike PROX1, MAFA is expressed in mature β-cells and plays a major role in the regulation of insulin secretion [21]. The *PROX1* variant is very rare (Table 1), and the *MAFA* variant has not been described in the literature or databases yet. Both variants cause amino acid exchanges. Bioinformatics tools consistently predict pathogenic significance for the *MAFA* variant Thr57Arg, while prediction for the *PROX1* variant c.1180G>A, p.Ala394Thr, is predominantly benign (Table 1). A further classification of the variants according to the ACMG (American College of Medical Genetics) [22] criteria was based on multiple sequence alignments and extensive functional data from the literature (Table 1).

**Figure 6:**
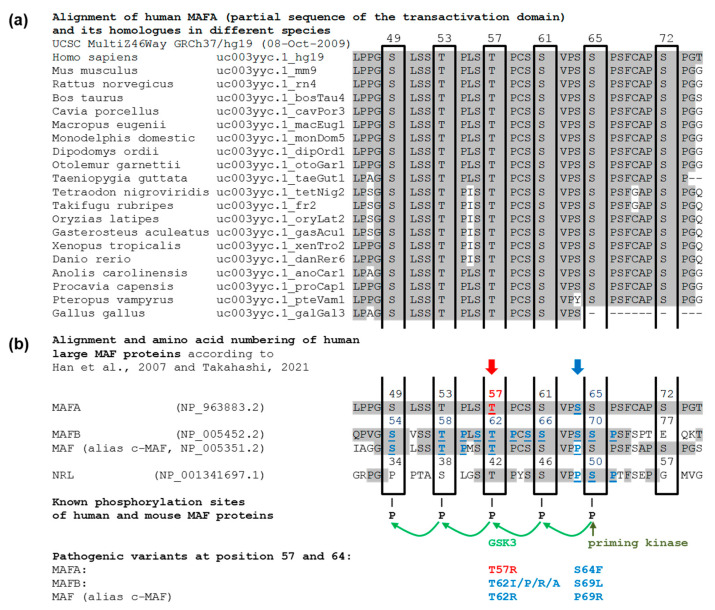
D/E/S/T/P-rich region of the MAFA transactivation domain (**a**) illustrates high conservation of a functionally essential part of the MAFA transactivation domain known to regulate MAFA binding to its target DNA sequences by phosphorylation. Conserved amino acid residues are shaded gray, and black frames indicate the phosphorylation sites. The numbering refers to human MAFA. (**b**) shows the high homology of this region among the members of the large MAF transcription factor family in humans and illustrates the GSK3-mediated phosphorylation cascade, which is initiated by the phosphorylation of residue 65 (MAFA) by a priming kinase (21, 23). Amino acid residues known to be affected by pathogenic variants are provided in blue and underlined, and the MAFA variant Ser64Phe is among them (blue arrow, S64F), which has been shown to cause a phosphorylation defect and a syndrome characterized by insulinomatosis, mild diabetes, and cataract (12). The red arrow marks the amino acid affected by the MAFA variant Thr57Arg (T57R) reported in this manuscript. In vitro transfection studies (23) have confirmed that the exchange of MAFA residues 65, 61, 57, 53, and/or 49 by alanine, an amino acid which cannot be phosphorylated, causes phosphorylation defects and impairs MAFA-binding to DNA. For two further large MAF proteins (MAFB and c-MAF), pathogenic missense variants have already been detected and among them include the MAFB variants Thr62Arg (21) and Thr62Ala (ClinVar VCV000807627.3, T62I/P/R/A) and the MAF (c-MAF) variant Thr62Arg (T62R) (24), variants with the same amino acid exchange as we now found in MAFA.

The MAFA variant is located in a region of the transactivation domain that is highly conserved between species (Figure 6a) and among the members of the large MAF transcription factor family (Figure 6b). The phosphorylation of this D/E/S/T/P-rich region is known to be crucial for binding MAFA to its target DNA sequences [23,24,25,26], regulation of β-cell insulin secretion [13,27], transforming activity of MAFA [28], and MAFA degradation by the proteasome [23,25,29,30,31]. Thr57 is one of the four serine and threonine residues phosphorylated in a series of phosphorylation steps mediated by GSK3 (Glycogen synthase kinase 3) and initiated primarily by phosphorylation of Ser65 by a priming kinase (Figure 6b). A set of evidence from in vitro and in vivo studies has confirmed that this basal mechanism is very similar among human large MAF proteins [21]. Transfection studies in which the five crucial serine and threonine residues of MAFA were systematically replaced by alanine show a significant phosphorylation defect for all constructs, including the Thr57Ala construct [23]. Apart from *MAFA* translocations in patients with multiple myeloma, only one pathogenic variant in the *MAFA* gene is known, Ser64Phe. It prevents phosphorylation of Ser65 by the priming kinase. Ser64Phe leads to a characteristic syndrome in which some family members, mainly women, are affected by insulinomatosis and other family members, mainly men, by mild diabetes [12], consistent with the observations in the family described here. No benign variants have been reported within this part of the transactivation domain. In the large databases (e.g., dbSNP and GnomAD), single, extremely rare variants of uncertain significance are listed, but none of them affects phosphorylated residues 49, 53, 57, 61, and 65. However, variants in homologous residues of other large MAF proteins have been described, and they are all pathogenic. In both, MAFB and c-MAF (alias MAF), a Thr62Arg variant (corresponding to Thr57Arg in MAFA) causes characteristic syndromes with autosomal dominant inheritance. MAFB Thr62Arg and several other amino acid exchanges at this position cause Multicentric Carpotarsal Osteolysis Syndrome (MCTO, OMIM #166300) [21,32], and Thr62Arg in c-MAF results in the Ayme-Gripp syndrome (OMIM #601088) [24] (Figure 6b). Interestingly, the clinical picture caused by MAFB and c-MAF variants depends on their localization. Variants that are not located in the highly phosphorylated part of the transactivation domain cause different syndromes, respectively. Based on this evidence and in accordance with the ACMG criteria, we classified the MAFA variant Thr57Arg as pathogenic even at this stage of investigation. In contrast to that, the detected PROX1 variant is not located in one of the essential domains. Several PROX1 variants have been associated with a risk for alterations in glucose and lipid metabolism [33], but not with monogenic diabetes or insulinomatosis. For these reasons and because we had already classified the MAFA variant as pathogenic, we assessed the PROX1 variant as likely benign, even though it cannot be ruled out as a modifier of the disease.

#### 3.2.2. Molecular Genetic Testing of Asymptomatic Family Members

Four generations underwent further molecular genetic and laboratory testing as well as an evaluation of their medical history (Figure 1, Table 2). In addition to the index patient and her sister, a total of 17 clinically asymptomatic family members were investigated (eight female and nine male). Apart from the two index cases, *MAFA* variant Thr57Arg was detected in 10 other family members (five female and five male). None of them had been diagnosed with insulinomatosis so far. In the entire family, however, four members were already diagnosed with overt type 2 diabetes (three men and one female). Two of them (III3 and IV2; Figure 1) had died from other causes (pancreatic cancer and cancer of unknown primary): One is treated with insulin and metformin (III1), and one is treated with diet only (V3). Two further family members (one female and one male) showed borderline HbA1c and/or impaired fasting glucose (IFG) levels (V2, HbA1c 5.8% and V5, IFG, HbA1c 5.9% [ref.: 4.1–5.6%]; Figure 1, Table 2).

Four patients presented with a tumor disease (one with lung cancer (IV1), one with lung cancer and a brain tumor (III1), one with pancreatic cancer (IV2), and one with a cancer of unknown primary (III3)), and one female with advanced age has dementia. There was no history of congenital eye disorders in this family.

Genetic testing revealed that the six symptomatic (insulinomatosis or mild diabetes; classification here after laboratory analyses) individuals studied from generations III-V (Figure 1, III1; IV3, 4; V2, 3, 5) all carried the *MAFA* variant, but only three of them had the *PROX1* variant (IV3, 4; V5). Generation VI and individuals III2 and V1 could not be analyzed in this regard because only a buccal swab was available for genotyping, but no EDTA blood was available for determining HbA1c. In the six healthy subjects studied, the MAFA variant was absent (III4, 5; IV5; V4, 6, 7), whereas the *PROX1* variant was detected in two of them (III4, V4). Thus, the segregation of these variants in the family clearly suggests the *MAFA* variant as the cause of the disease and further autosomal dominant inheritance.

## 4. Discussion

Using whole exome sequencing, we identified a so far unreported missense *MAFA* variant (c.170C>G, p.Thr57Arg) as the cause of insulinomatosis in two sisters with recurrent hyperinsulinemic hypoglycemia. Thr57Arg is located in MAFA’s highly conserved transactivation domain, which is so crucial that extensive functional analyses have already been performed. These include expression studies confirming that the replacement of threonine at position 57 with an amino acid that cannot be phosphorylated by GSK3 results in a defect in the stepwise phosphorylation of MAFA, impairing the binding of MAFA to its DNA target and preventing proteasome-mediated degradation [23,25,29,30,31]. So far, only one other pathogenic *MAFA* variant (c.191C>T, p.Ser64Phe) has been described. It is located in the same MAFA domain, impairs the same phosphorylation cascade, and results in the same characteristic symptoms in the two affected families as observed in the family described here [12]. In summary, we describe a novel pathogenic *MAFA* missense variant that is highly likely to cause impaired phosphorylation and, therefore, functional defects, cosegregating with both phenotypes, insulinomatosis and MODY-like diabetes (“MODY-like”: autosomal dominantly inherited, rather mild hyperglycemia (diabetes/IFG/HbA1c > 5.6) caused by a pathogenic variant in a transcription factor regulating insulin secretion). The detection of this second pathogenic *MAFA* variant confirms that a phosphorylation defect in the transactivation domain is accompanied by a characteristic set of symptoms in the affected family and confirms the autosomal dominant inheritance of the disease.

Iacovazzo et al. recently demonstrated for the first time that the Ser64Phe mutation of the *MAFA* gene was linked to insulinoma or mild, non-insulin dependent diabetes [12]. Ser64Phe was discovered to impair phosphorylation within MAFA’s transactivation domain and to profoundly increase MAFA protein stability in β-cell-lines at high and low glucose concentrations. There was no significant change in the amount of wild-type and mutant *MAFA* mRNA in transfected cells, confirming that the effect on protein turnover was posttranscriptional. These findings imply that the activity of Ser64Phe-MAFA is enhanced in β-cells/pancreas islets due to an increased transactivation capacity that is at least partly related to decreased MAFA degradation [12,23,25,29,30,31]. However, the transactivation activity of MAFA can be both decreased and increased depending on the cell type studied and the expression of other transcription factors [12,23,25,28,29,30,31]. The Thr57Arg *MAFA*-variant found in our family, similar to that reported by Iacovazzo et al. [12], is located within the highly conserved D/E/S/T/P-rich domain, impairing phosphorylation within MAFA’s transactivation domain, thus enhancing the protein stability and probably the total transactivation capacity of MAFA. Whether a phosphorylation defect in the D/E/S/T/P-rich domain of MAFA increases or decreases, net MAFA protein expression in β-cells cannot be conclusively answered at present. Both Iacovazzo et al. [12] and our group found no obvious difference by immunohistochemistry between MAFA expression in islets and insulinomas in insulinomatosis patients and islets from patients not affected by insulinomatosis, but the data are based on very few cases so far.

MAFA, MAFB, and MAF are large MAF proteins that have been shown to be oncogenes in tissue cultures, animal models, and human cancer. MAFA’s ability to induce proliferation of quail neuroretina cells [34] and chicken embryo fibroblasts [35] when overexpressed demonstrated its transformation potential. Large MAF proteins are overexpressed in 50% of multiple myelomas and 60% of angioimmunoblastic T-cell lymphomas in humans. In particular, in 8–10% of multiple myelomas the *MAF*, *MAFB*, and *MAFA* genes are translocated to the immunoglobulin heavy chain locus [36]. When expressed alone in β-cells, MAFA is only a weak transactivator of the insulin promoter, but when co-expressed with Pdx1 and Beta2/NEUROD1, these three factors synergistically and strongly activate the promoter [27]. The simultaneous expression of these three factors in non-β-cells (e.g., liver cells) induced the expression of the endogenous insulin gene, as well as other important β-cell genes (*GCK*, *SLC2A2*, *G6PC2*, *KCNJ11*, and *SUR1*) [23,29,30]. Thus, these three key β-cell transcription factors can be used for trans-differentiating non-β-cells into insulin-producing cells [37]. Immunohistochemical examinations in patients with *MEN1* associated insulinomas demonstrated a linked decrease in both menin and MAFA. Moreover, in mouse insulinomas, decreased MAFA expression resulting from targeted *MEN1* ablation was consistently observed [38]. Furthermore, in vitro analysis using insulinoma-derived cell lines revealed that menin regulates MAFA on both protein and mRNA levels and binds to *MAFA* promoter sequences. Menin knockdown concomitantly decreased the mRNA expression of both *MAFA* and β-cell differentiation markers (*INS*, *GCK*, *SLC2A2*, and *PDX1*) while increasing tumor proliferation, indicating that altered menin expression disrupts the MAFA differentiation pathway in insulinoma.

In conclusion, there is growing evidence that mutations of *MAFA*, which impair phosphorylation within the transactivation domain, result in increased MAFA protein stability and probably transactivation capacity. The transactivation activity exerted by MAFA in this process is presumably dependent on the maturity and degree of differentiation of the β-cell or its progenitor, as well as on the expression of further transcription factors and possibly also on the current metabolic conditions. This mechanism could explain the physiological properties of MAFA acting as an oncogene as well as a key transcription factor of islet β-cells, which regulates insulin secretion and the function of differentiated β-cells.

However, the mechanisms explaining how the same gene variant causes either diabetes or insulinomatosis are still unclear. Iacovazzo et al. [12] discussed a similarly paradoxical phenotype described for pathogenic variants of the transcription factor *HNF4A* and the potassium channel gene *ABCC8*, in which diabetes is preceded by transient congenital hyperinsulinism in some patients [39]. In accordance with the results of Iacovazzo et al. [12], the two phenotypes appeared to be mutually exclusive in the family described here, even though we cannot comment on the glycemic status of the two patients prior to developing insulinomatosis. All mutation–positive patients with available glycemic status had insulinomatosis or diabetes/impaired glucose tolerance. The pedigree (Figure 1) clearly confirms the previously reported [14] autosomal dominant mode of inheritance as well as the high penetrance of the disease (insulinomatosis or mild impairment of glucose metabolism). Family members with hyperglycemia had a mean BMI of 27.2 kg/m^2^ (excluding one outlier (V2) with BMI of 38), with a slight preponderance of the male gender. Diabetes was managed through diet and in one case by insulin administration (III1). There were no significant diabetic complications reported by family members. In contrast to the family members with hyperglycemia, both patients with insulinomatosis were females, which is consistent with the findings of Iacovazzo et al. [12] demonstrating that insulinomatosis is predominant in females. Additionally, sporadic insulinomas are more common in females too (male-to-female ratio of 1:1.4).

Several mechanisms are proposed by Iacovazzo et al. [12] as possible explanations for this phenomenon. In vitro, the treatment of human β-cells and human insulinomas with estrogens has been shown to enhance proliferation and insulin secretion [40]. Furthermore, during pregnancy, an expansion of β-cell mass induced by prolactin and placental lactogen signaling mediated by the prolactin receptor (PRLR) is observed. PRLR was significantly downregulated and the *PRLR* promoter was shown to be directly activated by MAFA in luciferase reporter assays in both *MAFA* knockout islets and MIN6 β-cells with siRNA-mediated knockdown of MAFA [41]. Therefore, estrogens and prolactin could potentially promote β-cell proliferation, predisposing female carriers of *MAFA* mutations to insulinomatosis. Similarly to the family members with insulinomatosis reported by Iacovazzo et al. [12], one of our female insulinomatosis patients experienced the first hypoglycemic symptoms after pregnancy. She did not complain about any clinical problems during the pregnancy. The other affected sister, however, had never been pregnant and developed the first symptoms after puberty. In summary, it is striking that there is a female preponderance for developing insulinomatosis and a male preponderance for developing diabetes/impaired glucose tolerance in the three families with *MAFA* mutations reported so far. However, the precise mechanisms that determine the development of either insulinomatosis or diabetes remain unknown, and it is not ruled out that diabetes and insulinomatosis may develop consecutively. Furthermore, it is not excluded that the *PROX1* variant detected in our family acts as a modifier in this regard.

In clinical practice, diagnosis and therapy of familial insulinomatosis remains challenging. In large studies, insulinomatosis is responsible for less than 5% of all patients with hyperinsulinemic hypoglycemia [2,10]. There are no distinct clinical or biochemical characteristics that distinguish solitary insulinoma from multifocal insulinomatosis. Multiple insulin-secreting microtumors are present in affected patients, distributed throughout the entire pancreas and only detectable by histological examination. Even when utilizing modern functional imaging modalities such as Glucagon-like peptide-1 receptor (GLP-1R) imaging, only few macrotumors with a mean size of 0.5–1 cm can be detected [42,43]. Thus, the clinical course of the disease is still characterized by early recurrent hypoglycemia requiring repeated surgical intervention. All documented clinical cases had persistent or recurrent disease after the initial surgical intervention. Only the histological examination of a larger, representative pancreatic specimen confirmed the diagnosis of insulinomatosis and distinguished it from multifocal NET with *MEN1* mutations or nesidioblastosis. The SACI test should be considered in cases where noninvasive imaging modalities are negative or when there is evidence of a multifocal disease, as it can distinguish solitary insulinoma from multifocal/diffuse diseases with sensitivity and specificity of 95–100% and 90%, respectively, and determine the extent of surgical intervention [44,45].

The mean age at the diagnosis of insulinomatosis in our family was 39.5 years, being almost exactly the same as reported in the families by Iacovazzo et al. [12] (39.4 ± 13.1 years). Unfortunately, we were unable to determine the exact age at the diagnosis of diabetes. However, the recorded past medical history of the living family members indicated that the initial presentation of diabetes in our family occurred between 29 (V5) and 50 years of age (compared to 38.4 ± 16.4 years as reported by Iacovazzo et al. [12]). Therefore, most patients with *MAFA* mutations show clinical features of insulinomatosis or diabetes already in young adulthood. In contrast, the youngest patients with *MAFA* mutations and diagnosed diabetes or insulinomatosis were 11 and 18 years old, respectively [12]. We, therefore, recommend that asymptomatic mutation carriers should be monitored for diabetes/impaired glucose tolerance beginning in adolescence and that these patients should receive regular clinical screening as well as information/counselling, especially for signs and symptoms of neuroglycopenia.

The findings of this study have to be seen in light of some limitations. A more detailed characterization of the mild hyperglycemia observed in four variant carriers (Figure 1) could provide further insights in the dynamics of the glucose metabolism in these patients. The affected family members consented to a one-time examination (including blood glucose and HbA1c), but not to an oral glucose tolerance test. Furthermore, it would be valuable to assess the glycemic status of the variant carriers in generation VI (genotyping by buccal swab). The parents were educated about potential symptoms of hypoglycemia and hyperglycemia, but they declined blood sampling and functional testing of the asymptomatic children at that time.

While it is clear that Thr57Arg causes a phosphorylation defect, which increases protein stability, and while Thr57Arg probably increases MAFA-mediated transactivation capacity of the beta cell, the consequences for MAFA’s transactivation activity per molecule remain largely unknown. Using the MAFA Thr57Ala construct, transactivation activity has already been studied extensively by others. As the result was clearly dependent on cell type, differentiation, and embryonic or neoplastic origin of the cell model, it did not seem reasonable to repeat the expression studies on another (or the same) cell models only replacing Thr57Ala by Thr57Arg. Extensive further studies are needed to elucidate the complex regulation of MAFA’s transactivation activity.

## 5. Conclusions

We define a second *MAFA* variant, p.Thr57Arg, as the cause of familial insulinomatosis by linking genetic, bioinformatic, clinical, biochemical, and family data to functional in vitro analyses from the literature. We, thus, confirm MAFA phosphorylation defects as an important cause of a hereditary syndrome, which is characterized by insulinomatosis and/or mild, MODY-like hyperglycemia. This study extends the pathophysiological and diagnostic disease concept and verifies the high penetrance and autosomal dominant inheritance pattern of familial insulinomatosis.

## Figures and Tables

**Table 1:** Comparison and classification of the potentially disease-causing variants in *PROX1* and *MAFA* genes detected by WES and variant filtering.

	*PROX1*	*MAFA*
Genotype	GA	CG
Mutation Call	c.1180G>A	c.170C>G
Amino Acid Change	p.Ala394Thr	p.Thr57Arg
Exon	1	1
Coverage	101	103
Chromosome	1	8
RefSeq	NM_001270616.2	NM_201589.4
dbSNP	rs1217787927	no entry
ClinVar	no entry	no entry
GnomAD	1/251,260 alleles	no entry
Mutation Taster	disease causing	disease causing
PolyPhen2	benign	probably damaging
SIFT	tolerated	damaging
PROVEAN	neutral	deleterious
OMIM Gene	601546	610303
OMIM Disease	no entry	147630
Inheritance (OMIM)	no entry	AD
ACMG criteria	likely benign	pathogenic

ACMG = American College of Medical Genetics.

**Table 2:** Clinical characteristics and laboratory results of two patients with insulinomatosis (IV3 and IV4) and five relatives (V2, 3, 4, 5; III4). In family members III5, IV5, V6, and V7, the laboratory tests indicated in the table were also performed. Since there were no abnormalities in glucose metabolism and neither the *MAFA* nor the *PROX1* variant was detected in the corresponding branch of the family, we have not presented the test results in this table.

	Reference	Patient IV3	Patient IV4	Patient V5	Patient V3	Patient V2	Patient V4	Patient III4
**Sex/age**	-	F/57	F/48	M/29	M/38	F/30	M/31	F/81
**BMI**	18.5–24.9 kg/m^2^	27.0	26.0	26.6	29.0	38.0	29.9	26.1
**Preexisting diagnosis**	-	Insulinomatosis Diabetes Depression	Insulinomatosis Diabetes Hypothyroidism Crohn’s disease	Allergic asthma Impaired fasting glucose (IFG)	Allergic asthma Diabetes Rosacea	-	Allergic asthma Hypothyroidism Gastro-oesophageal reflux disease (GERD)	Hypertension Atrial fibrillations Cataract-operation Polyarthritis
**Reported therapy**	-	Duodenum-preserving resection of the pancreatic head (2010)	Left-sided pancreatectomy (2008) Tumor-enucleation of the pancreatic head (2009) Duodenum-preserving subtotal pancreatectomy (2010)	-	-	-	-	-
**Medication**	-	Pancreatic enzymes Pantoprazole	Insulin-therapy Pancreatic enzymes	Cetirizine	Formoterol/Beclomethasone	Oral contraceptives	Cetirizine Omalizumab Levothyroxine	Candesartan Bisoprolol Rivaroxaban
**MAFA**	c.170C>G, p.Thr57Arg	C/G	C/G	C/G	C/G	C/G	C/C	C/C
**HbA1c**	4.1–5–6%	6.9 ↑	7.5 (↑)	5.9 (↑)	6.6 ↑	5.8 (↑)	5.4	5.4
	21–38 mmol/mol Hb	51	59	42	49	40	35	36
**F-Glucose**	70–100 mg/dL	143 ↑	128 ↑	129 ↑	125 ↑	95	94	85
**Insulin**	6–25 mU/L	6.7	6.3 *	24.3 ↑	26.5 ↑	15.7	9.3	4.5
**Pro-insulin**	<9.4 pmol/L	8.07	<0.1 *	2.42	7.50	11.2 ↑	5.86	3.10
**C-Peptide**	0.8–5.2 ng/mL	2.0	<0.01 *	2.75	4.52	3.37	2.54	1.66
**OH-Butyrate**	<74 µmol/L	70	60	45	30	154 ↑	21	183
**CRP**	<5 mg/L	1.1	0.54	1.6	3.5	22 ↑	2.5	3.6
**TG**	<150 mg/dL	74	63	154 (↑)	260 ↑	138	261 (↑)	118
**Cholesterol**	<200 mg/dL	181	141	214 ↑	254 ↑	192	263 ↑	235 ↑
**HDL-C**	>40 mg/dL	81	75	55	45	71	56	82
**LDL-C**	<160 mg/dL	85	53	128	157	93	155	129
**AST**	5–31 U/L	25	34	24	73 ↑	27	34	29
**ALT**	<35 U/L	21	27	40	171 ↑	19	59 ↑	14
**ALP**	37–111 U/L	103	98	65	87	57	90	106
**GGT**	9–36 U/L	24	14	29	78 ↑	11	59	19
**Bilirubin**	0.3–1.2 mg/dL	0.58	0.42	0.49	0.45	0.38	0.59	0.54
**Albumin**	35–50 g/L	40	38	40	41	37	46	41
**Lipase**	<60 U/L	12	4	38	36	29	30	41

BMI body mass index; CRP = C-reactive protein; TG = Thyroglobulin; HDL-C = high-density lipoprotein cholesterol; LDL-C = low-density lipoprotein cholesterol; AST = aspartate aminotransferase; ALT = alanine aminotransferase; ALP = alkaline phosphatase; GGT = gamma glutamyl transferase; ↑ = increased values compared to reference range; (↑) = marginal increased values compared to reference range; * = insulin therapy.

## Data Availability

All relevant data generated or analyzed during this study are included in this published article. Restrictions apply to the availability of the complete next generation sequencing data of the patients to preserve patient confidentiality. The corresponding author will on request detail the restrictions and any conditions under which access to some data may be provided.
